# Chemical characterization of commercial liquid smoke products

**DOI:** 10.1002/fsn3.9

**Published:** 2013-01-08

**Authors:** Naim Montazeri, Alexandra CM Oliveira, Brian H Himelbloom, Mary Beth Leigh, Charles A Crapo

**Affiliations:** 1 Kodiak Seafood and Marine Science, Center School of Fisheries and Ocean Sciences, University of Alaska FairbanksKodiak, Alaska, 99615; 2 Institute of Arctic Biology, Department of Biology and Wildlife, University of Alaska, FairbanksFairbanks, Alaska, 99775

**Keywords:** GC-MS, Gibbs reaction, liquid smoke, phenol content

## Abstract

The objective of this study was to determine important chemical characteristics of a full-strength liquid smoke, Code 10-Poly, and three refined liquid smoke products (AM-3, AM-10 and 1291) commercially available (Kerry Ingredients and Flavors, Monterey, TN). The pH of the products were significantly different (*P* < 0.05) and ranged from 2.3 (Code 10-Poly) to 5.7 (1291). The pH was inversely correlated with titratable acidity (*R*^2^ = 0.87), which was significantly different (*P* < 0.05) among products ranging from 10.3% acetic acid (Code 10-Poly) to 0.7% acetic acid (1291). Total phenol content was quantified using the Gibbs reaction; the only liquid smoke containing appreciable level of phenolic compounds was Code 10-Poly at 3.22 mg mL^−1^. Gas chromatography-mass spectrometry (GC-MS) analysis of liquid smoke dichloromethane extracts revealed that carbonyl-containing compounds were major constituents of all products, in which 1-hydroxy-2-butanone, 2(5H)-furanone, propanal and cyclopentenone predominated. Organic acids were detected by GC-MS in all extracts and correlated positively (*R*^2^ = 0.98) with titratable acidity. The GC-MS data showed that phenolic compounds constituted a major portion of Code 10-Poly, and were detected only in trace quantities in 1291. The refined liquid smokes had lighter color, lower acidity, and reduced level of carbonyl-containing compounds and organic acids. Our study revealed major differences in pH, titratable acidity, total phenol content, color and chemical make-up of the full-strength and refined liquid smokes. The three refined liquid smoke products studied have less flavor and color active compounds, when compared with the full-strength product. Furthermore, the three refined products studied have unique chemical characteristics and will impart specific sensorial properties to food systems. Understanding the chemical composition of liquid smokes, be these refined or full-strength products, is an important step to establish their functions and appropriate use in food systems.

## Introduction

Liquid smokes have been used extensively in food systems to impart flavor characteristics that are similar to smoked food products (Varlet et al. 2010). These may be used to preserve quality and ensure safety of foods (Schubring 2008; Martin et al. 2010). Liquid smokes are usually obtained from the condensation of wood smoke produced by smoldering wood chips or sawdust under limited oxygen. Commercial full-strength liquid smokes are commonly fractionated, purified and concentrated to yield aqueous, oil or dry powder products. Through the refining process, undesirable polycyclic aromatic hydrocarbons (PAH) are removed, and the intensity of flavor and color in the resulting refined liquid smoke is adjusted (Cadwallader 2007; Varlet et al. 2010). Refined liquid smokes generally offer more flexible applications to particular food systems when compared with full-strength liquid smoke products.

The chemical composition of liquid smokes depends primarily on the wood type and moisture content of wood, the latter influences the pyrolysis temperature and the duration of smoke generation (Guillen and Ibargoitia 1999; Cadwallader 2007). A majority of the dry mass of wood is composed of cellulose, hemicelluloses and lignin. Thermal decomposition of cellulose produces anhydroglucose, carbonyl-containing compounds and furans. Hemicellulose decomposition is similar to cellulose decomposition, but yields acetic acid and carbon dioxide. Partial pyrolysis of lignin produces various types of phenolic compounds (Miler and Sikorski 1990). Therefore, the thermal degradation of wood results in a complex mixture of compounds, which characterize the overall organoleptic, antioxidative and antibacterial properties of full-strength liquid smokes (Guillen and Manzanos 1999a; Milly et al. 2005; Wei et al. 2010).

Baltes et al. (1981) found the major proportion of commercial full-strength liquid smoke to be composed of water (11–92%), tar (1–17%), acids (2.8–9.5%), carbonyl-containing compounds (2.6–4.6%) and phenol derivatives (0.2–2.9%). However, in the manufacturing of liquid smokes, a variety of ingredients may be used, such as salts, fatty acids, fatty esters and carriers like saccharides (Guillen and Manzanos 1997; Varlet et al. 2010). Phenolic compounds contribute to smoke flavor and color of liquid smokes, and also have antibacterial and antioxidant properties (Clifford et al. 1980; Maga 1987; Varlet et al. 2010). Carbonyl-containing compounds impart sweet or burnt-sweet aroma and tend to soften the heavy smoky aroma associated with phenolic compounds with some “typical smoke-cured” aroma and flavors (Fujimaki et al. 1974; Kim et al. 1974; Kostyra and Barylko-Pikielna 2006). Furthermore, carbonyl-containing compounds are involved in textural changes in smoked food caused by interaction with proteins, and contribute the golden-brown color of smoked products due to reaction with amino acids, and the formation of Maillard reaction products (Varlet et al. 2007).

Liquid smokes, either full-strength or refined, offer several advantages over wood smoke, such as better control of PAH content, wider diversity of applications to food systems, superior product homogeneity, easiness to store and less environmental pollution (Varlet et al. 2010). A disadvantage of full-strength liquid smokes is their high content of flavor and color active compounds, which limit their application when used for specific purposes such as antimicrobial agents (Vitt et al. 2001). As stated, full-strength liquid smokes can be refined for various applications. The desired aroma and color characteristics of the final product are achieved by adjusting the content of phenol derivatives, carbonyl-containing compounds and organic acids (Kim et al. 1974; Underwood 1992; Toledo 2007). It has been demonstrated that commercial liquid smoke preparations are effective against various types of spoilage and pathogenic microorganisms (Milly et al. 2005). However, at times it may be challenging to ensure that the liquid smoke used for a particular application will deliver the desired final sensorial properties of a food product. For instance, liquid smokes with high content of carbonyl-containing compounds are generally applied to food systems as browning agent (Underwood 1992), while refined liquid smokes containing reduced levels of color and flavor active compounds are applied as color preservatives in raw tuna and salmon (Schubring 2008) or as an antimicrobial additive in frankfurters (Martin et al. 2010).

Commercial production and fractionation of liquid smokes involve proprietary or patented information; thus, the specific chemical constituents of these commercial products are generally not disclosed to buyers. This poses difficulty for food product developers to determine appropriate levels and types of liquid smoke products to be used for specific applications in food systems. Establishing the chemical composition of commercial liquid smokes is the first step to better understand their interaction with the chemical components of food systems. It may also be advantageous in predicting the resulting sensorial properties of the final product. These are two key points to define the functions and appropriate uses of liquid smokes in food systems.

The objective of this study was to determine important chemical characteristics of a full-strength liquid smoke, Code 10-Poly, and three refined liquid smoke products (AM-3, AM-10 and 1291) commercially available (Kerry Ingredients and Flavors, Monterey, TN). The chemical volatile and semi-volatile constituents of these products were identified using gas chromatography-mass spectrometry (GC-MS) analysis. Color, pH, titratable acidity and total phenol content were also determined.

## Material and Methods

### Procurement and handling of liquid smokes

A quantity of 2 L each of full-strength liquid smoke (Code 10-Poly lot no. 0831038703) and of three refined liquid smoke fractions (AM-3 lot no. 09009038703, AM-10 lot no. 0910038701 and 1291 lot no. 0316038820) were procured from Kerry Ingredients and Flavors in June 2010. Manufacturer information for these preparations indicated that the three refined liquid smokes with less color and smoky flavors could be applied to foods as antibacterial and/or shelf-life-enhancing additives with minimized undesirable sensorial impacts. These refined liquid smokes are produced using different processes except AM-10 which is a refined product from AM-3 (M. van der Bleek, Kerry Ingredients and Flavors, pers. comm.). Each product was transferred immediately from the plastic containers to 1 L glass jars covered with two layers of aluminum foil and stored at 4°C until analysis. Code 10-Poly was analyzed along with the refined liquid smokes to compare the extent of refining process on final product characteristics. Analyses were conducted within 2 weeks of sample procurement.

## Color

The Gardner Delta Color Comparator (BYK Additives and Instruments, Columbia, MD) was used to determine the color of duplicate liquid smoke samples. The comparator is equipped with two wheels embedded with nine glass filters with colors ranging from colorless through deep amber. The colors correspond to the Gardner color values ranging from 1 to 18. According to the manufacturer's instruction, about 10 mL of a liquid sample was placed in a glass tube, which was positioned between the two wheels. The wheels were rotated until the filter glass closest in color to the liquid was in place and the filter notation was recorded.

## pH and titratable acidity

An aliquot of 5 mL (ca. 5 g) was sampled from each liquid smoke, and pH at 25°C was recorded with a SevenEasy pH meter (Mettler Toledo, Schwerzenbach, Switzerland) equipped with a probe (model WD-35801-00; Oakton Instruments, Vernon Hills, IL). For titratable acidity (TA) (as % acetic acid), the liquid smokes were diluted (1:4 w/v) in deionized water and titrated to pH 8.3 using 0.1 *N* NaOH (J. T. Baker Chemical Co., Phillipsburg, NJ) as described by Sadler and Murphy (2003). All pH measurements were conducted in triplicate.

## Extraction of volatile and semi-volatile compounds in liquid smokes

The commercial liquid smokes were subjected to liquid–liquid extraction as described by Guillen and Manzanos (1999a) with minor modifications. A sample of 25 mL of each liquid smoke was extracted with 50 mL dichloromethane (Honeywell Burdick & Jacksons, Muskegon, MI) in a 100 mL two-neck round-bottom flask (Chemglass, Vineland, NJ) fitted with a thermometer. The upper neck of the flask was fitted with a water-jacketed reflux condenser with an internal coil-type cold finger (Kimble Chase, Vineland, NJ) connected to a recirculating cooler (Model F 12, Julabo, Allentown, PA) kept at 6.5°C. Another end of the condenser was open to the atmosphere. The flask was heated in an oil bath for 7 h at 40°C and stirred with a magnetic stirrer bar. A 12.5 mL aliquot of the extract was transferred to a 20 mL capped amber glass vial (Agilent Technologies, Wilmington, DE), and 4 g of anhydrous Na_2_SO_4_ (Mallinckrodt Chemicals, Phillipsburg, NJ) was added. After 4 h, the extract aliquot was centrifuged (5 min, 3,000 rpm) and the liquid portion carefully transferred, using a glass Pasteur pipette, to another 20 mL capped amber glass vial. The extract volume was reduced at 25°C using a gentle stream of research-grade nitrogen gas. The final volume for each of the three refined liquid smoke extracts (AM-3, AM-10 and 1291) was 1 mL, a tenth of the volume suggested by Guillen and Manzanos (1999a). In the case of Code 10-Poly, it was not possible to reduce its dichloromethane extract to 1 mL because constant volume was achieved at 4.5 mL; therefore, this was its final volume.

## Separation, identification and qualification of volatile and semi-volatile compounds in liquid smokes

A gas chromatograph (GC, Model 6890; Agilent Technologies) interfaced with a mass spectrometer (MS, Model 5973; Agilent Technologies) equipped with a DB-5HT capillary column (30 m × 0.25 mm × 0.1 *μ*m, Model 122–5731; Agilent Technologies) was used. Helium was used as a carrier gas at 1.0 mL min^−1^ of flow and average velocity of 36 cm s^−1^. The GC was operated in constant flow mode. The nominal inlet pressure and temperature were 7.67 psi and 150°C, respectively. The transfer line temperature was kept at 280°C. The injection volume was 1 *μ*L at 1:100 inlet split ratio. The Code 10-Poly extract was exactly diluted with dichloromethane to 1:10 (v/v) because gas chromatography of a 1 *μ*L aliquot of the dichloromethane extract that had been reduced to a constant volume of 4.5 mL, injected at 1:100 inlet split ratio, yielded misshaped chromatographic peaks displaying significant front-tailing, poor symmetry and extensive overlaps of contiguous peaks. Differently, the three refined liquid smoke fractions (AM-3, AM-10 and 1291) were analyzed using 1 *μ*L aliquots of their dichloromethane extracts that had been reduced to a final volume of 1 mL, corresponding to one tenth of the volume suggested by Guillen and Manzanos (1999a). When the refined liquid smoke extracts were reduced to a final volume of 10 mL (Guillen and Manzanos 1999a), fewer peaks were observed at much lower abundances. Initial oven temperature was 50°C and held for 0.5 min, followed by an increment of 2°C min^−1^ until 250°C, and a final hold time of 15 min. The total run time was 115.5 min. The MS was operated in electron impact ionization mode at 70 eV and acquired data after a solvent delay of 2 min. The mass range for acquisition was 50–300 amu at a scan rate of 5.02 scans s^−1^. The temperature of the MSD transfer line, source and quadrupole were 280°C, 230°C and 150°C, respectively. Compounds were identified using the National Institute of Standards and Technology'98 (NIST'98) library (Agilent Technologies). When the MS spectra quality (MSQ) was >90%, the compound was considered “likely known,” when between 80% and 90% MSQ as “tentatively identified” and when MSQ <80% as “unknown.” Peak areas were recorded and reported as total ion count (TIC) and as peak percentage (% TIC), while retention times were recorded and reported in minutes.

## Total phenol content

Nicholson (1984) method was used to measure total phenol content (TPC) in liquid smokes. This method is a modification of Tucker (1942) procedure based on the Gibbs (1927) method that quantifies the intensity of a colorimetric reaction of the Gibbs reagent with phenolic compounds. The stock Gibbs reagent was prepared by dissolving 0.25 g of 2,6-dichloroquinone-4-chloroimide (Sigma-Aldrich, St. Louis, MO) in 30 mL of absolute alcohol (Spectrum, Gardena, CA). The stock solution is stable for several months if kept in a cold and dark environment (Svobodova et al. 1978). A working solution, to be used immediately, was prepared by adding 1 mL of the stock solution to 15 mL of deionized water (nanopure). A buffer was prepared by mixing 125 mL of 0.4 *M* boric acid (J. T. Baker Chemical Co., Phillipsburg, NJ), 125 mL of 0.4 *M* (Sigma-Aldrich, Steinheim, Germany) and 40 mL of 0.2 *M* NaOH (Fisher Scientific, Fair Lawn, NJ), and bringing the mixture volume to 1 L using deionized water. The liquid smokes were diluted with deionized water to 1:40, 1:400 and 1:4,000 (v/v, liquid smoke: deionized water).

A sample of 5 mL of liquid smoke diluted in deionized water and five phenol solutions with concentrations of 3, 6, 9, 12 and 15 μg mL^−1^ phenol (Sigma-Aldrich, St. Louis, MO) were each mixed with 5 mL of pH 8.3 buffer. Deionized water was used as blank. A quantity of 1 mL of 0.15 *N* NaOH was added followed by 1 mL of the Gibbs working solution. The mixture was kept in the dark for 25 min at 25°C. Indophenols result from the reaction as indicated by blue color development (Gibbs 1927). The absorbance was recorded at 635 nm using a Cary 50 UV–Visible Spectrophotometer (Varian Inc., Walnut Creek, CA) and the TPC calculated using a calibration curve linear to 15 μg mL^−1^ phenol (*R*^2^ = 0.996). The TPC was determined in each of the liquid smoke solutions in triplicate (Nicholson 1984).

## Statistical analyses

Linear regression and correlation were performed using Microsoft® Excel 2011 (Ver. 14.2.3: Microsoft Corporation, Santa Rosa, CA) to establish standard curve for TPC measurement and to evaluate the statistical relationships among TA, pH and organic acid content (*α* = 0.05).

## Results and Discussion

### Color

Freshly prepared liquid smokes were bright yellow; however, color changes quickly take place because some of the smoke components condense or polymerize rendering the mixture brown (Guillen and Manzanos 1997, 1999a, 1999b; Simko 2005). The four liquid smokes studied showed no visible turbidity or precipitate formation during 2 months of storage at 4°C. The Code 10-Poly had the darkest Gardner color value of 16, followed by AM-3, AM-10 and 1291 with values of 11, 5 and 2, respectively. The Gardner Delta Color Comparator is a simple, inexpensive, and non-destructive instrument that is used to measure color of vegetable oil (Cermak and Isbell 2002 and fish oil (U.S. Food and Drug Administration 2003). Limited information is available in the literature regarding the instrumental quantification of liquid smoke color. Our results demonstrated that this instrument shows potential in an industry setting for quick measurement of color in liquid smoke products.

## pH and titratable acidity

Acids influence flavor (tartness), color, texture and microbial stability of food (Hollenbeck 1977; Sadler and Murphy 2003; Rozum 2007). Acidity of liquid smokes depends on the wood source, processing steps and refining parameters (Guillen and Ibargoitia 1999; Rozum 2007; Sung et al. 2007; Toledo 2007). Liquid smokes are usually acidic with a pH of 1.5–5.5 (Toth and Potthast 1984); however, an alkaline liquid smoke (pH = 7.7) prepared from black tea leaves is reported by Sung et al. (2007) These authors observed that the pH of bread flour liquid smoke was 4.2, and when combined with black tea, the pH of resulting liquid smoke increased to 5.5 (Sung et al. 2007. The Code 10-Poly had the highest acidity with pH of 2.3 and TA (% acetic acid) of 10.3 (mean ± 0.0 SD, *n* = 3). On the other hand, the refined ones had a higher pH and lower TA. The pH and TA of AM-3, AM-10 and 1291 were 4.3, 4.2, and 5.7, and 2.2, 2.3, and 0.7 (% acetic acid), respectively (mean ± 0.0 SD, *n* = 3). In the liquid smoke manufacturing process, acids may be neutralized to decrease the harshness of smoke flavor (Toledo 2007). This change in pH may affect food texture, color development and flavor intensity (Maga 1987; Fiddler et al. 1970). Among the product tested, 1291 had the highest pH and lowest TA, which indicates a probable neutralization step during manufacturing, and less acidic flavor change to food.

The pH of the four products investigated were inversely correlated (*R*^2^ = 0.87; TA = −2.99(pH)+16.17) to TA values and similar to the results reported by Milly et al. (2005). However, a correlation between organic acid content and pH is not observed for liquid smokes prepared from different woods (Chen and Maga 1993). Similar results are observed in wine (Plane et al. 1980) and milk (Walstra et al. 2006). The lack of correlation between the two parameters could be attributed to differences in dissociation constants of the organic acids present in the products. A pH measurement reflects the quantity of hydronium ions (H_3_O^+^) in the media; however, not all organic acids may be dissociated (Sadler and Murphy 2003). Conversely, TA is based on the ability of an alkaline titrant to dissociate weak organic acids (Sadler and Murphy 2003). Based on our results, TA appeared to be a better predictor of acidity in liquid smokes. With respect to organic acids impacts on flavor, a combination of hydrogen ions, anions and/or protonated acid species dictates acid taste (Plane et al. 1980; Sadler and Murphy 2003; Neta et al. 2007).

## Volatile and semi-volatile compounds in liquid smokes

Dichloromethane is a widely used solvent to extract the chemical constituents of liquid smokes (Edye and Richards 1991; Guillen et al. 1995, 2001; Sung et al. 2007). Dichloromethane has a low boiling point (40°C) and extracts aromatic compounds efficiently (Guillen and Manzanos 1999a). Some drawbacks of using a dichloromethane extraction is the escape of low boiling point <40°C compounds (Guillen et al. 2001) and partitioning of very polar compounds, such as short-chain organic acids, between the liquid smoke aqueous phase and the dichloromethane phase. Furthermore, there are non-volatiles and particulate matter that may contribute to smoke flavor but do not extract with dichloromethane (Maga 1987).

**Figure 1:**
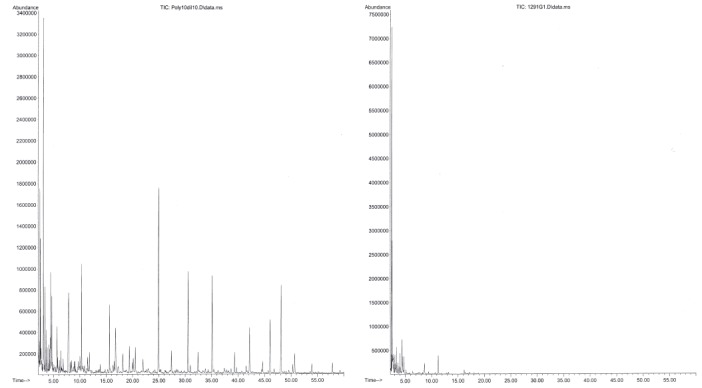
Selected chromatograms of Code 10-Poly (left) and 1291 (right) at 1:100 inlet split ratio, where abundance is reported as the total ion count (TIC) and time in minutes (min).

The 1:100 inlet split ratio analysis provided better separation and peak resolution for the majority of detected compounds, but was not sufficient to identify some compounds present at low abundance. Therefore, all four extracts were also injected using a 1:50 inlet split ratio to investigate the possible identity of certain peaks (Tables 1 and 2 footnote). Selected chromatograms of Code 10-Poly and 1291 dichloromethane extract, as two extreme examples of a full-strength and a refined liquid smoke, are represented in Figure 1. Phenolic compounds are an important group of smoke constituents responsible for smoke flavor notes in liquid smokes and in smoked food products. Phenols also have antibacterial and antioxidant properties (Maga 1987). Phenolic compounds were only detected in the Code 10-Poly extracts and in trace quantities in the 1291 extracts. Carbonyl-containing compounds (aldehydes, ketones, furan and pyran derivatives) constituted a major portion of all four extracts. This class of compounds provides odor and flavor background notes described as sweet, caramel and magi (instant broth), which rounds-up the smoky odor in liquid smokes (Kostyra and Barylko-Pikielna 2006), contributes to the browning coloration in smoked foodstuff (Varlet et al. 2007), and may display antibacterial properties (Milly et al. 2005).

**Table 1:** Volatile and semi-volatile compounds tentatively identified in dichloromethane fractions of Code 10-Poly

*t*_R_ (min)	Compounds (mass spectra data)	MSQ^1^	TIC^2^	%TIC	Other names^3^
Phenolic compounds					
6.38	Phenol	94	554.2	0.5	
9.08	2-methylphenol	95	350.0	0.3	*Ό*-cresol
10.02	4-methylphenol	92 (93)^4^	601.5	0.6	*ρ*-cresol
10.32	2-methoxyphenol	97	3,908.4	3.9	Guaiacol
13.51	Dimethylphenols	93	101.6	0.1	
14.56	4-ethylphenol OR 3-ethylphenol	62-70 (80)^4^	121.9	0.1	
14.78	2-methoxy-3-methylphenol	93 (94)^4^	179.0	0.2	
15.60	2-methoxy-4-methylphenol OR 2-methoxy-5-methylphenol	95-95	2,457.1	2.4	
16.79	1,2-benzenediol	91	3,319.3	3.3	Pyrocatechol; catechin
19.41	3-methoxy-1,2-benzenediol	95 (97)^4^	1,262.3	1.2	3-methoxypyrocatechol
20.15	3-methyl-1,2-benzenediol	95	846.5	0.8	3-methylcatechol
22.01	4-methyl-1,2-benzenediol	91 (93)^4^	751.8	0.7	4-methylcatechol
20.51	4-ethyl-2-methoxyphenol	90	1,281.3	1.3	4-ethylguaiacol
21.03	4-methoxy-3-methylphenol	83	184.2	0.2	
23.03	3-hydroxybenzaldehyde OR 4-hydroxybenzaldehyde OR 2-hydroxybenzaldehyde	96–90–81	346.0	0.3	Anisaldehyde;*m*-formylphenol;*ρ*-formylphenol;*Ό*-formylphenol (salicylaldehyde)
24.95	2,6-dimethoxyphenol	94	12,482.4	12.3	Syringol
25.17	Eugenol	98	206.3	0.2	*ρ*-allylguaiacol
27.39	Vanillin	81	1,043.2	1.0	2-hydroxy-3-methoxybenzaldehyde
28.64	4,5-dimethyl-1,3-benzenediol	59 (92)^4^	352.1	0.3	
31.06	2-methoxy-4-propylphenol	87	347.3	0.3	Cerulignol; 4-propylguaiacol
38.23	4-hydroxyacetyl-2-methylphenol	80	187.0	0.2	
39.34	2,6-dimethoxy-4-(2-propenyl) phenol	97 (98)^4^	858.2	0.8	4-allylsyringol
41.54	4-hydroxy-3-methoxybenzeneacetic acid	74 (81)^4^	431.6	0.4	(4-hydroxy-3-methoxyphenyl) acetic acid
42.21	4-hydroxy-3,5-dimethoxybenzaldehyde	90 (93)^4^	2,273.0	2.2	Syringaldehyde
	Total phenolic compounds		34,445.9	34.0	
Aldehydes and ketones					
2.60	Propanal	87	1,960.8	1.9	
2.44	1-hydroxy-2-butanone	87	2,710.9	2.7	
4.28	2-methyl-2-cyclopenten-1-one	94	398.2	0.4	
4.70	1,2-cyclopentanedione	87 (91)^4^	2,388.3	2.4	
4.80	2,5-hexanedione	87	315.5	0.3	
5.61	4-methyl-2-hydroxycyclopent-2-en-1-one	90	133.4	0.1	
5.81	1-(acetyloxy)-2-butanone	86 (91)^4^	452.6	0.4	
6.60	3,4-dimethyl-2-cyclopenten-1-one	95	220.1	0.2	
7.85	2-hydroxy-3-methyl-2-cyclopenten-1-one	94	5,459.4	5.4	Cyclotene
8.16	2,3-dimethyl-2-cyclopenten-1-one	89 (94)^4^	438.4	0.4	
8.95	3,4-dimethyl-2-hydroxycyclopent-2-en-1-one	95	416.7	0.4	
11.87	3-ethyl-2-hydroxy-2-cyclopenten-1-one	95	1,261.5	1.2	
13.85	2,3-dihydroxybenzaldehyde	95	320.8	0.3	
19.97	3,4-dihydroxyacetophenone	83 (87)^4^	390.2	0.4	1-(3,4-dihydroxyphenyl) ethanone
28.08	1-(3-hydroxyphenyl) ethanone	93	252.3	0.2	
37.47	2-ethoxy-4-anisaldehyde	80	365.9	0.4	
32.51	1-(4-hydroxy-3-methoxyphenyl) ethanone	97	965.6	1.0	Acetovanillone; 4-acetyl-2-methoxyphenol
45.71	4-hydroxy-2-methoxycinnamaldehyde	90 (95)^4^	135.8	0.1	(2E)-3-(4-hydroxy-2-methoxyphenyl)-2-propenal
46.04	1-(4-hydroxy-3,5-dimethoxyphenyl) ethanone	97	2,662.7	2.6	Acetosyringone; 4-acetyl-2,6-dimethoxyphenol
57.89	3,5-dimethoxy-4-hydroxycinnamaldehyde	93 (97)^4^	504.2	0.5	
	Total aldehydes and ketones		21,726.3	21.4	
Furans and pyrans					
2.88	Furan-3-carbaldehyde	91 (94)^4^	121.3	0.1	3-furfural; 3-furaldehyde
3.10	Furan-2-carbaldehyde	91	5,716.2	5.6	2-furfural; 2-furaldehyde
3.82	Tetrahydro-2,5-dimethoxyfuran	90	610.1	0.6	
4.38	1-(2-furanyl)-ethanone	94	387.6	0.4	Acetylfuran
4.42	Butyrolactone	90	513.1	0.5	2(3H)-furanone
4.48	2(5H)-furanone	91	2,735.6	2.7	2-butenolide; γ-crotonolactone
5.06	5-methyl-2(5H)-furanone	87 (90)^4^	175.6	0.2	β-Angelica lactone
5.68	5-methyl-2-furancarboxaldehyde	94	605.5	0.6	5-methylfurfural
6.05	Methyl furan-3-carboxylate	83	124.0	0.1	Methyl ester 3-furoic acid
6.41	3-methyl-2(5H)-furanone	91	426.7	0.4	2-methyl-2-butenolide
6.97	2,5-dihydro-3,5-dimethyl-2-furanone	91	548.2	0.5	
8.36	4-methyl-5H-furan-2-one	78 (87)^4^	732.1	0.7	4-methyl-2(5H)-furanone
11.51	Maltol	90 (93)^4^	757.6	0.7	3-hydroxy-2-methyl-4h-pyran-4-one
18.01	5-(hydroxymethyl)-2-furancarboxaldehyde	94	1,020.2	1.0	
	Total furans and pyrans		14,473.6	14.3	
Organic acids					
2.74	Butanoic acid	90	187.9	0.2	Butyric acid
3.25	2-butenoic acid	86	257.6	0.3	Crotonic acid
30.56	3-hydroxy-4-methoxybenzoic acid	80	4,999.2	4.9	Isovanillic acid
65.70	9-(E)-octadecanoic acid	99	460.8	0.5	Oleic acid
	Total organic acids		5,905.4	5.8	
Miscellaneous					
15.70	1,4:3,6-dianhydro-*α*-d-glucopyranose	83 (89)^4^	465.1	0.5	
17.89	2,3-anhydro-d-mannosan	90	521.9	0.5	
28.32	1,2,3-trimethoxy-5-methylbenzene	90 (92)^4^	330.2	0.3	3,4,5-trimethoxytoluene
29.84	3-hydroxy-benzoic acid methyl ester	83 (87)^4^	317.6	0.3	
34.19	1,4-dimethoxy-2-methylbenzene OR 3-isopropylthiophenol	83-80 (87-86)^4^	200.1	0.2	
34.36	4-hydroxy-3-methoxy-benzoic acid methyl ester	93 (94)^4^	136.8	0.1	Vanillic acid methyl ester
36.04	6-hydroxycoumarin	90 (93)^4^	154.4	0.2	6-hydroxy-2H-1-benzopyran-2-one
	Total miscellaneous		2,126.0	2.1	
	Total area of known compounds		78,677.3	77.6	
	Total area of unknown compounds with >2 %TIC		9,593.7	9.5	

1 MSQ, MS spectrum quality according to NIST98 library.

2 Code 10-Poly was injected at 1:10 dilution, the area of peaks are multiplied by 10, and reported in 10^4^ TIC.

3 Extracted from NIST Chemistry WebBook, NIST Standard Reference Database Number 69 (http://webbook.nist.gov/chemistry/name-ser.htm, accessed November 2011).

4 MSQ values in parentheses are for peaks when samples were injected at 1:50 inlet split ratio.

**Table 2:** Volatile and semi-volatile compounds tentatively identified in dichloromethane fractions of refined liquid smokes

		AM-3			AM-10			1291			
*t*_R_ (min)	Compounds (mass spectra data)	MSQ^1^	TIC^2^	%TIC	MSQ	TIC	%TIC	MSQ	TIC	%TIC	Other names^3^
Phenolic compounds											
20.15	3-methyl-1,2-benzenediol							81 (90)^4^	9.8	0.1	3-methylcatechol
	Total phenolic compounds		0.0	0.0		0.0	0.0		9.8	0.1	
Aldehydes and ketones											
2.60	Propanal	83	1,741.3	5.7	80 (90)^4^	1,409.8	8.2	80	693.1	6.3	
2.44	1-hydroxy-2-butanone	86 (90)^4^	2,342.5	7.6	86	1,920.9	11.2	87	5,020.5	46.0	
3.07	4-hydroxy-2-pentanone	64 (90)^4^	493.8	1.6	93	267.8	1.6	83	257.6	2.4	
3.15	2-cyclopenten-1-one	83	873.9	2.8	91 (93)^4^	166.7	1.0	93	98.4	0.9	
3.56	2-butanone	80	2,056.1	6.7	72 (80)	947.8	5.5				
4.80	2,5-hexanedione				90	116.4	0.7				
5.81	1-(acetyloxy)-2-butanone	90 (91)^4^	163.2	0.5				91	14.5	0.1	
5.96	3-methyl-2-cyclopenten-1-one	91	326.8	1.1							
6.83	1,2-cyclohexanedione	93	30.7	0.1							
7.85	2-hydroxy-3-methyl-2-cyclopenten-1-one	94 (95)^4^	1,004.8	3.3							Cyclotene
	Total aldehydes and ketones		9,033.0	29.3		4,829.4	28.2		6,084.1	55.7	
Furans and pyrans											
2.35	2-methoxytetrahydrofuran							90 (91)^4^	965	6.4	
2.63	Tetrahydro-2-furanol				80	19.4	0.1	87	103.7	0.9	
3.82	Tetrahydro-2,5-dimethoxyfuran							94	100.4	0.9	
3.92	Dihydro-2H-pyran-3(4H)-one	86	392.5	1.3	83 (86)^4^	195.4	1.1				3-tetrahydropyranone
3.93	Tetrahydro-2-furanmethanol				83	16.4	0.1				
3.98	Tetrahydro-2-methyl-2-furanol	80	29.6	0.1							
4.38	1-(2-furanyl)-ethanone	86 (87)^4^	185.1	0.6							Acetylfuran
4.42	Butyrolactone							91	437.4	4.0	2(3H)-furanone
4.48	2(5H)-furanone	94 (95)^4^	7,361.2	23.9	90 (95)^4^	3,767.7	22.0	91	243.9	2.2	2-butenolide; γ-crotonolactone
5.06	5-methyl-2(5H)-furanone	95	685.7	2.2	95	155.9	0.9				β-Angelica lactone
6.41	3-methyl-2(5H)-furanone	91	569.3	1.8	91 (93)^4^	65.4	0.4				2-methyl-2-butenolide
6.22	2H-pyran-2-one	93	14.6	tr							Coumalin
6.97	2,5-dihydro-3,5-dimethyl-2-furanone	91	270.2	0.9							
8.22	3,4-dimethyl-2,5-furandione							93	11.9	0.1	Dimethylmaleic anhydride
											
8.36	4-methyl-5H-furan-2-one	91	985.3	3.2							4-methyl-2(5H)-furanone
12.29	5-acetyldihydro-2(3H)-furanone				83	99.7	0.6				Solerone
15.57	5-hydroxymethyldihydrofuran-2-one				86	112.0	0.7				
18.01	5-(hydroxymethyl)-2-furancarboxaldehyde	94	3,277.9	10.6							
	Total furans and pyrans		13,771.4	44.7		4,431.9	25.8		1,591.9	14.6	
Organic acids											
2.29	Propanoic acid	90	197.0	0.6	90	414.0	2.4	90	175.1	1.6	Propionic acid
3.04	3-butenoic acid							90	13.8	0.1	
3.25	2-butenoic acid				93	20.2	0.1		64.0	0.6	Crotonic acid
11.42	4-oxo-pentanoic acid	80	124.9	0.4							Levulinic acid; 4-oxo-valeric acid
65.70	9-(E)-octadecanoic acid				93	44.1	0.3				Oleic acid
	Total organic acids		321.8	1.0		478.3	7.4		252.9	2.3	
Miscellaneous											
3.08	2-propenyl-butanoate				83	787.5	4.6				
11.91	Glycocyanidine				80	265.3	1.5				
15.70	1,4:3,6-dianhydro-.alpha.-d-glucopyranose	83	140.8	0.5	93	1,248.5	7.3				
17.89	2,3-anhydro-d-mannosan	93	1,408.9	4.6	86 (93)^4^	725.2	4.2				
	Total miscellaneous		1,549.6	5.0		3,026.5	17.6				
	Total area of known compounds		24,675.8	80.1		12,766.1	74.4		7,938.7	*72.7*	
	Total area of unknown compounds with peak area >2 %TIC		2,125.9	6.9		1,532.3	8.9		548.5	*5.0*	

1 MSQ, MS spectrum quality according to NIST98 library.

2 Area in 10^4^ TIC.

3 Extracted from NIST Chemistry WebBook, NIST Standard Reference Database Number 69 (http://webbook.nist.gov/chemistry/name-ser.htm, accessed November 2011); tr, %TIC <0.1.

4 MSQ values in the parentheses are for peaks when samples were injected at the 1:50 inlet split ratio. Note: The blank cells for TIC and %TIC indicate that the chemicals were not detectable.

## Phenolic compounds

Several phenolic compounds were detected in the Code 10-Poly and accounted for 34% TIC (Table 1). Identification of phenol was confirmed by injecting a phenol standard solution (Sigma-Aldrich) into the GC-MS along with the sample analysis (data not shown).

Syringol (2,6-dimethoxyphenol) was the most abundant phenolic compound, followed by guaiacol (2-methoxyphenol), and pyrocatechol (1,2-benzenediol). The ratio of syringol derivatives to guaiacol derivatives was approximately 1.2:1 and lower than 3.3:1, which is considered an indicator that hardwood was used as a wood source (Baltes et al. 1981). When softwood is used to produce smoke, syringol derivatives are barely detectable (Edye and Richards 1991; Guillen et al. 1995). Our results indicated a mixture of hardwood and softwood might have been used to produce Code 10-Poly. Guaiacol and pyrocatechol are antioxidants found in smoke (Fujimaki et al. 1974; Guillen et al. 1995, 2001; Maga 1987). Along with 4-methylguaiacol and syringol, guaiacol contributes to overall smoky flavor and odor (Wasserman 1966). Syringol and its derivatives are not associated with odor notes typically detected in freshly smoked food products (Kostyra and Barylko-Pikielna 2006). The relative ratio of the total content of guaiacol and its derivatives to that of phenol, cresols and xylenols may impart a considerable effect on the variability in the aroma of the phenolic fractions of wood smoke (Fujimaki et al. 1974). 4-methylphenol (*ρ*-cresol) and 2-methylphenol (*Ό*-cresol) are present in Code 10-Poly and are important contributors to the typical, palatable smoke-curing profile in liquid smoke product (Kostyra and Barylko-Pikielna 2006). Pyrocatechol is another important phenol in Code 10-Poly with antioxidant property that contributes to a heavy burnt and phenolic odor and some sweetness in liquid smokes (Fujimaki et al. 1974; Guillen et al. 1995, 2001). Among the refined liquid smokes, 3-methyl-1,2-benzenediol was only detected in trace quantities in 1291.

## Aldehydes and ketones

The refining process to produce AM-10 from AM-3 reduced the content of aldehydes and ketones in AM-10 (Table 2). The most refined 1291 contained the highest proportion of aldehydes and ketones at about 56% TIC. The most abundant compound was 1-hydroxy-2-butanone, which was also the major constituent of the other two refined liquid smokes. The 1-hydroxy-2-butanone is detectable in ether extracts of refined liquid smoke prepared from the branches of walnut tree (*Juglans* sp.) (Wei et al. 2010) and a commercial liquid smoke preparation (Charsol, Red Arrow Products Corp., Milwaukee, WI) (Fiddler et al. 1970). Formation of 1-hydroxy-2-butanone occurs during low temperature pyrolysis of wood hemicelluloses (Yi-min et al. 2009). The aroma perception of 1-hydroxy-2-butanone has a sweet, musty, coffee and grain-like odor (Kaseleht et al. 2011). Its isomer 3-hydroxy-2-butanone contributes to the pleasant butter-like odor of cold-smoked Atlantic salmon (*Salmo salar*) and as an indicator of spoilage (Jonsdottir et al. 2008).

Propanal was detected in all liquid smoke extracts, but was least abundant in 1291 (Tables 1 and 2). This aldehyde is detected in liquid smoke from grapevine shoots (*Vitis vinifera* L) and beechwood (*Fagus sylvatica* L) (Guillen and Ibargoitia 1996), but is not detected in a commercial Spanish liquid smoke (Guillen and Ibargoitia 1998). The 2-cyclopenten-1-one and its derivatives are usually found in wood or smoke in various amounts (Maga 1987; Guillen and Ibargoitia 1999) and have the organoleptic characteristics described as bitter taste and odor with grassiness and slight effect on smoke flavor (Kim et al. 1974). The 2-cyclopenten-1-one was absent in Code 10-Poly, but most of the methyl and dimethyl derivatives were detected (Table 1). Cyclotene (2-hydroxy-3-methyl-2-cyclopenten-1-one) was found in Code 10-Poly and AM-3 and detectable in AM-10 and 1291 only when samples were analyzed using an inlet split ratio of 1:50 (data not shown). These compounds are found usually in wood smoke or liquid smoke (Maga 1987; Guillen et al. 1995; Guillen and Manzanos 1996; Guillen and Ibargoitia 1999). Their organoleptic characteristic is described as bitter taste and grass odor notes (Kim et al. 1974). Similar to *ρ-*cresol and *Ό*-cresol, cyclotene mainly contributes to liquid smoke odor intensity and is characterized as a palatable taste typical of smoked foods (Kostyra and Barylko-Pikielna 2006). This ketone is detected in Atlantic salmon treated with liquid smoke and contributes to a potent “cooked/spicy” odor in the product (Serot et al. 2007).

## Furans and pyrans

Furans and pyrans result from thermal breakdown of cellulose and hemicellulose (Maga 1987) and contribute overall smoky odor to liquid smokes that soften partially the heavy smoky aroma of phenolic compounds (Kim et al. 1974). Their formation is attributed to Maillard reactions (Varlet et al. 2007). Furans are considered a secondary group of odor-active compounds, after phenolic compounds, in smoked Atlantic salmon extract (Serot et al. 2007). Pyrans impart green and roasty odor notes to salmon treated with liquid smoke (Serot et al. 2007). Furans and pyrans comprised about 26% and 45% TIC of AM-10 and AM-3, respectively (Table 2). The predominant compound was 2(5H)-furanone (2-butenolide), and its isomeric methyl derivatives were detected in three of the liquid smokes extracts studied but not in the 1291 extract. Furfurals (furan-2-carbaldehyde and furan-3-carbaldehyde) were detected only in Code 10-Poly and were in agreement with the chemical composition of liquid smoke prepared from grapevine shoot and beechwood (Guillen and Ibargoitia 1996), and walnut tree branches (Wei et al. 2010). Organoleptic characteristics of furfural are described as sweet, bread-like and caramel-like (Guillen and Ibargoitia 1996), and are linked with development of smoke odor in smoked foods (Serot et al. 2007; Toledo 2007). Detected in Code 10-Poly and AM-3, 5-methyl-2-furancarboxaldehyde imparts sweet fragrant and floral odor notes contributing to partial softening of the heavy smoky aroma of phenolic compounds in liquid smokes (Kim et al. 1974). Maltol was found only in Code 10-Poly and is one of the main furans detected in beechwood liquid smoke (Guillen and Ibargoitia 1999) and present in considerable amounts in several other liquid smoke products (Guillen and Ibargoitia 1996; Guillen and Manzanos 1997 1999a). Maltol is a chemical reference to “sweet odor” for sensory evaluation and training of panelists and has higher flavor intensity than furfurals (Ojeda et al. 2002). Other minor compounds in this group were 2(3H)-furanone in 1291 and Code 10-Poly and 4-methyl-5H-furan-2-one in AM-3 and Code 10-Poly (Tables 1 and 2).

## Organic acids

Organic acids result from the partial pyrolysis of wood cellulose and hemicellulose (Gilbert and Knowles 1975). During fish smoking, a range of organic acids may be deposited on the product surface (Guillen and Errecalde 2002). Organic acids are known for their impact on flavor (tartness), color, texture and microbial stability of food (Hollenbeck 1977; Sadler and Murphy 2003; Rozum 2007). Among these, acetic and lactic acids are added to food products for preservative (Baltes et al. 1981) and antibacterial purposes (Doores 1993). Butyric, caproic, capric and enanthic acids are mainly strong aroma carriers (Baltes et al. 1981). However, none of these organic acids were detected in our samples and was probably due to their high polarity and low affinity to dichloromethane. Similarly, acetic acid is not quantifiable in the dichloromethane extract of liquid smoke obtained from thyme (*Thymus vulgaris* L.) (Guillemet and Manzanos 1999a). However, acetic acid is found in liquid smoke obtained from beechwood (Guillen and Ibargoitia 1999) and the most abundant organic acid in liquid smoke prepared from a mixture of ponderosa pine (*Pinus ponderosa*) and cottonwood (*Populus trichocarpa*) (Edye and Richards 1991) and walnut tree branches (Wei et al. 2010).

Propanoic acid has a slightly pungent odor that contributes to the overall odor of liquid smokes and has antibacterial activity against spore-forming bacteria and molds in food (Doores 1993; Guillen and Manzanos, 1999a). This was the predominant organic acid in AM-3, AM-10 and 1291 (Table 2). Propanoic acid was a component of liquid smoke prepared from thyme (Guillen and Manzanos, 1999a). However, only trace amounts are detectable in liquid smoke from a mixture of ponderosa pine and cottonwood (Edye and Richards 1991). In Code 10-Poly, 3-hydroxy-4-methoxybenzoic acid was the predominant organic acid and may have been formed by secondary oxidation of aldehydes (Edye and Richards 1991).

The organic acid content in the liquid smoke products studied followed the values determined for TA and pH, as found by others (Edye and Richards 1991; Guillen and Ibargoitia 1996). The pH and TA of all products investigated were inversely correlated (*R*^2^ = 0.87). A good linear correlation (*R*^2^ = 0.98; TA = 2 × 10^−7^[organic acid content] + 1.17) was established between organic acids content and TA. However, a low correlation (*R*^2^ = 0.77; pH = −4 × 10^−8^[organic acid content] + 4.86) was observed for pH and organic acid content. Unlike pH, titratable acidity correlates well with the content of acetic acid (Edye and Richards 1991). Results indicated that TA was a more reliable parameter than pH for determining organic acid content of liquid smokes, and may these be full-strength or refined.

## Other compounds

Miscellaneous compounds such as sugars, benzene and esters were found in AM-3 and AM-10 (Table 2). Through pyrolysis, cellulose hydrolyzes to glucose followed by dehydration to 1,6-anhydroglucose (beta-glucosan) (Simon et al. 2005) and may explain its presence in liquid smokes. A third fragmentation produces acetic acid and its homologs (Simon et al. 2005) that may also be present in liquid smokes. The 1,2,3-trimethoxy-5-methyl-benzene was detected only in the Code 10-Poly and is detected at low concentrations in smoked Atlantic salmon treated with liquid smoke (Serot et al. 2007). Its characteristic odor descriptions are cooked and earthy notes (Serot et al. 2007).

In addition to the advantage of 1:50 inlet split ratio injection in enhanced identification of some compounds, it improved the detection of other compounds that were not detected at 1:100 inlet split ratio injection. Among carbonyl-containing compounds, 4-methyl-5H-furan-2-one was detected in AM-10 with 93% MSQ, and 5-hydroxymethyl-2-furancarboxaldehyde in 1291 with 94% MSQ. The ester 2-propenyl-butanoate was detected in 1291 with 83% MSQ. Pyrazine derivatives were not found in any samples analyzed, and these compounds contribute to a background smoky aroma associated with hickory smoke of liquid smokes (Maga and Chen 1985; Guillen and Manzanos, 1999a).

## Total phenol content

The calibration curve of phenol standards was linear for 3–15 *μ*g mL^−1^ (*R*^2^ = 0.997, absorbance = 0.077[phenol] + 0.009). The absorbance values for the phenol standard solutions ranged from 0.21 to 1.22. Blue color developed only with Code 10-Poly, which had a TPC of 3.22 ± 0.03 mg mL^−1^. This value was much lower than the phenol content reported in an aqueous fraction of liquid smokes prepared from mixed dried hardwood sawdust, which is in the range of 9.9–11.1 mg mL^−1^ (Ramakrishnan and Moeller 2002). The GC-MS analysis of the dichloromethane extracts (Table 2) revealed that neither AM-3 or AM-10 contained phenolic compounds, while 1291 contained only a trace amount of 3-methyl-1,2-benzenediol (0.1% TIC) and was below the minimum phenol concentration of 0.5 × 10^−4^ mg mL^−1^ required for blue color formation from indophenols (Gibbs 1927).

## Conclusion

This study determined key product characteristics of three commercially refined liquid smokes and one full-strength liquid smoke. The refining of liquid smokes eliminated phenolic compounds and, to a lesser extent, reduced contents of carbonyl-containing compounds and organic acids. As a result, the refined liquid smoke fractions AM-3, AM-10 and 1291 had significantly lower acidity, lighter color, and rather distinct chemical make-up when compared with the full-strength Code 10-Poly. The observed differences demonstrate that knowledge of the chemical characteristics of liquid smoke preparations is important to better understand their interactions with food components. Lower levels of carbonyl-containing compounds, as determined in the refined liquid smokes, may result in fewer changes to the original color and texture of the food product. Concomitantly, removal of phenol derivatives from liquid smokes may lower their impact in the final flavor of the food product. Nevertheless, prediction of impacts of liquid smokes in the organoleptic characteristic of food products is difficult because of the possibility of synergistic and antagonistic effects liquid smoke components may have with specific constituents of the food matrix. Overall, the results from this study are useful to food product developers seeking to determine a suitable liquid smoke product, and its appropriate level of use for application in a particular food system.

## Acknowledgments

This publication is the result of research sponsored by USDA-CSREES (grant no. 2008-34385-19314) and Alaska Sea Grant with funds from the National Oceanic and Atmospheric Administration Office of Sea Grant, Department of Commerce, under grant no. NA10OAR4170097 (project no. E/142-01), and from the University of Alaska Fairbanks with funds appropriated by the state. The authors thank Mark van der Bleek (pers. comm.) for providing the liquid smoke samples and valuable consultations.

## Conflict of Interest

None declared.
